# Supplementary figures and images for: Engineered extracellular vesicles enriched with the miR‐214/199a cluster enhance the efficacy of chemotherapy in ovarian cancer
Source: Mol Oncol. 2026 Feb 13:10.1002/1878-0261.70224. Online ahead of print. doi: 10.1002/1878-0261.70224 (PMC13398902; doi:10.1002/1878-0261.70224)

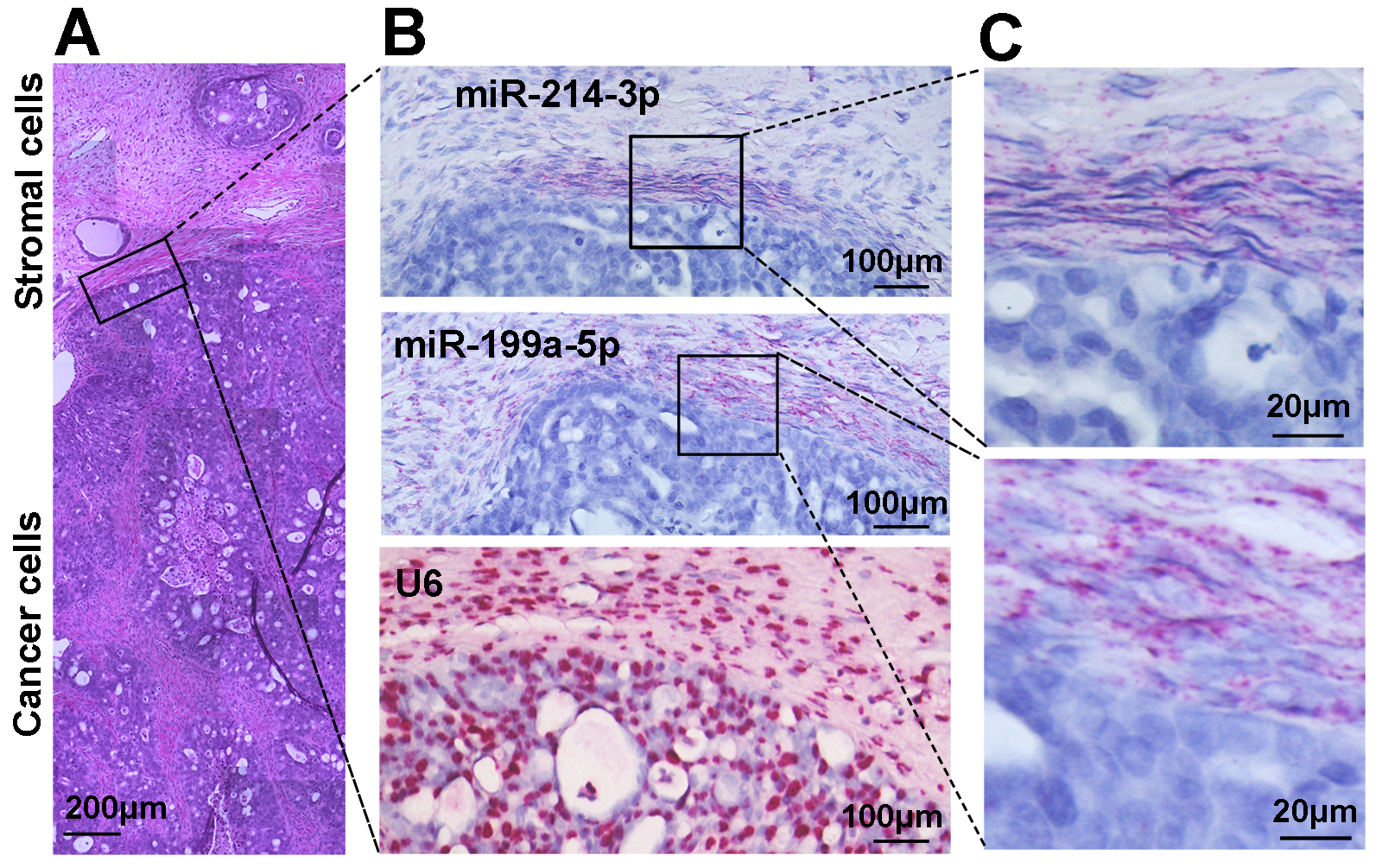

Supplement: Supplementary file 1 — Fig. S1. In situ detection of miR‐214‐3p and miR‐199a‐5p reveals stromal enrichment in recurrent HGSOC. [file MOL2-9999-0-s005.tif]

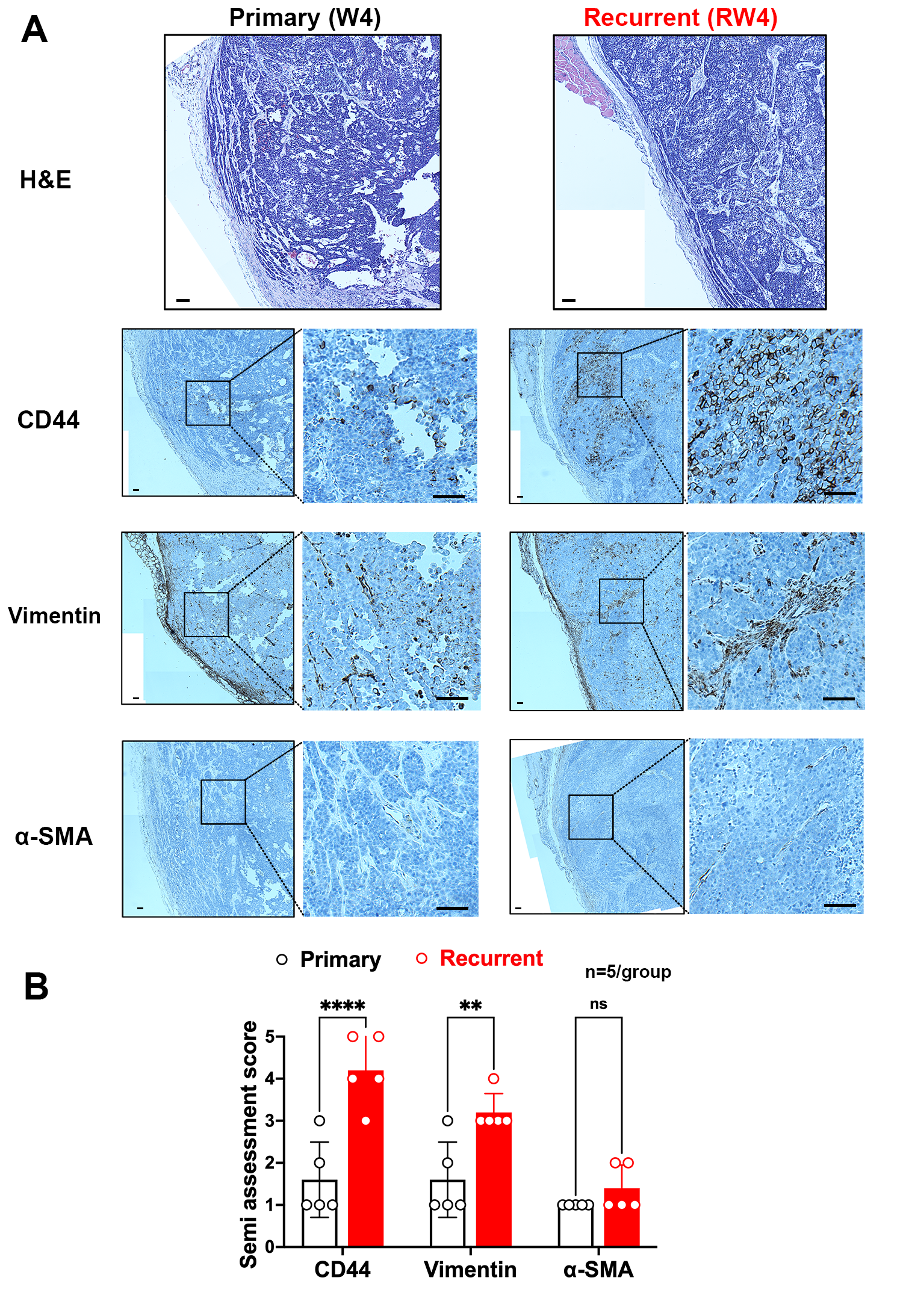

Supplement: Supplementary file 2 — Fig. S2. Immunohistochemical characterization of primary and recurrent ovarian cancer tissues. [file MOL2-9999-0-s007.tif]

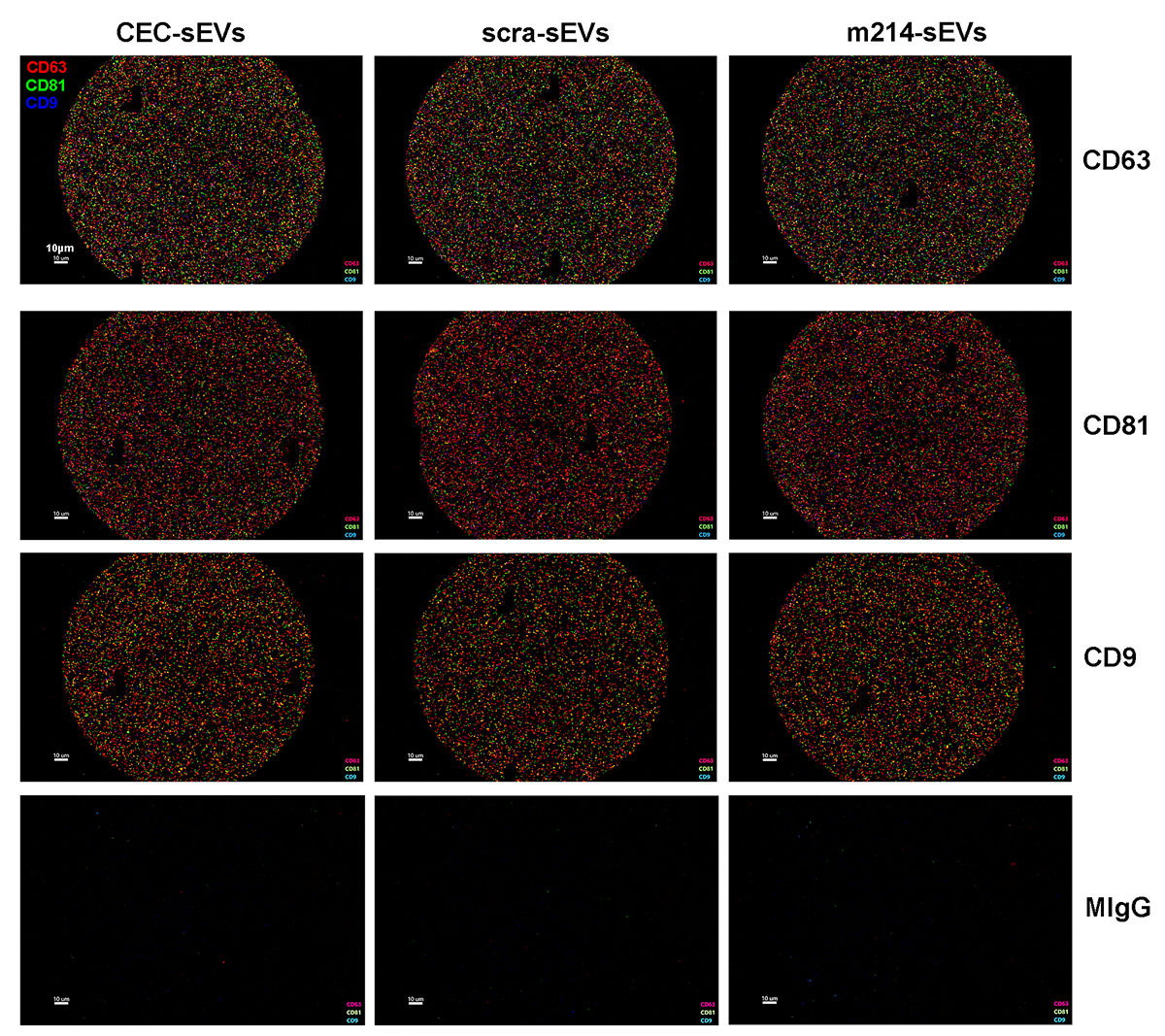

Supplement: Supplementary file 3 — Fig. S3. Single EV profiling using the ExoView system. [file MOL2-9999-0-s003.tif]

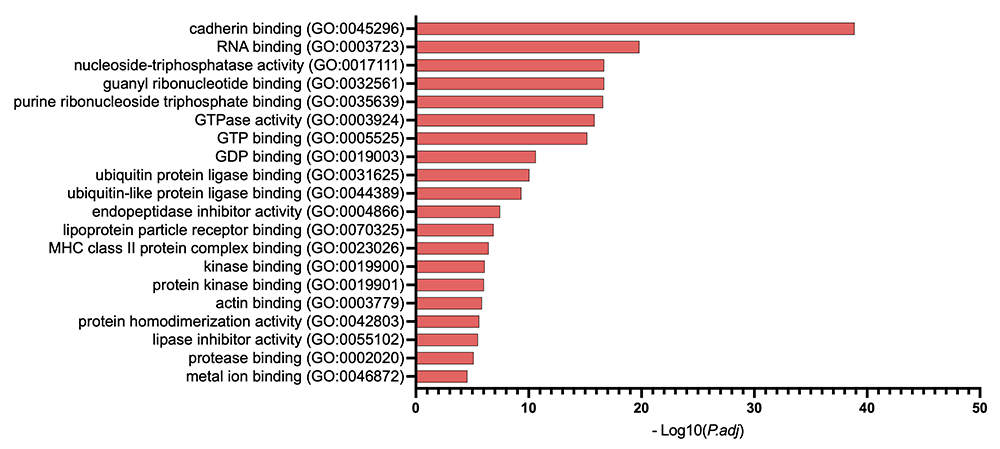

Supplement: Supplementary file 4 — Fig. S4. Top 20 enriched pathways associated with proteins in naïve CEC‐sEVs and m214‐sEVs. [file MOL2-9999-0-s008.tif]

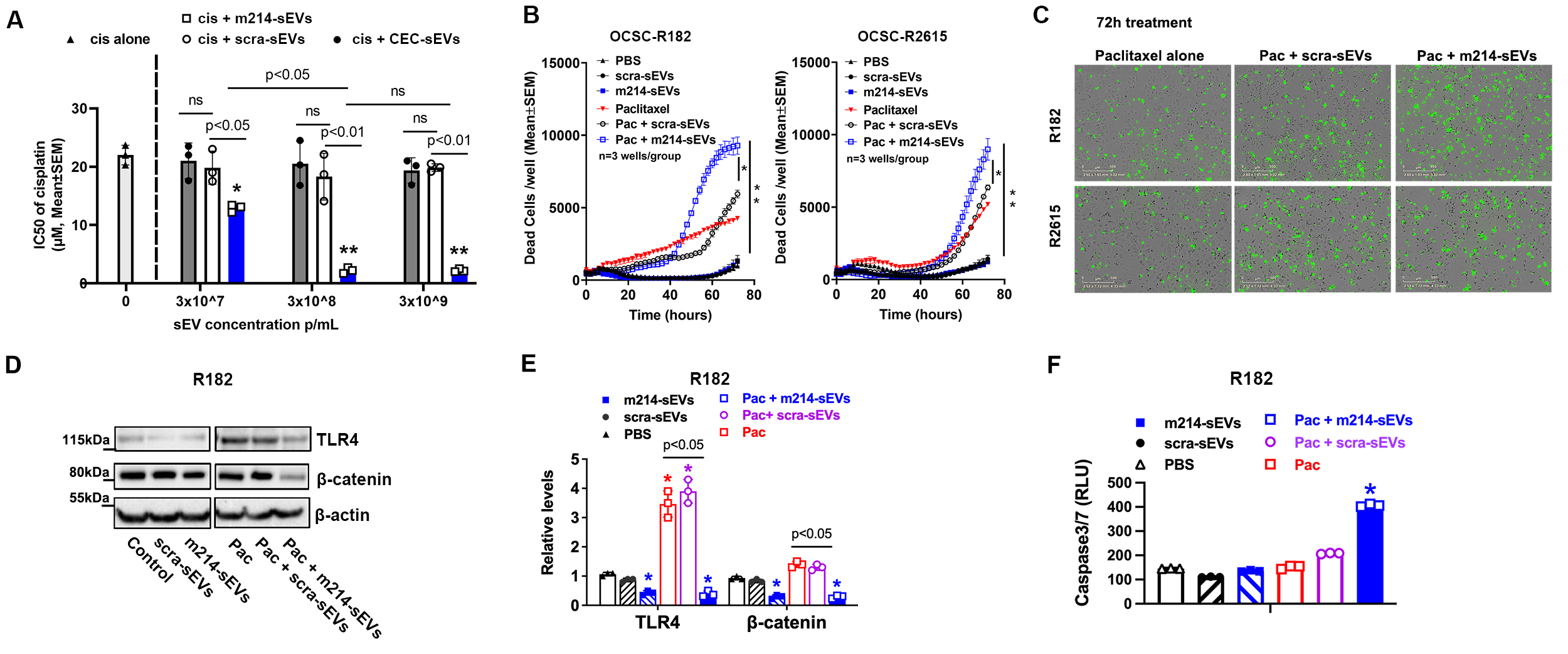

Supplement: Supplementary file 5 — Fig. S5. m214‐sEVs sensitize chemoresistant OC cells to cisplatin and paclitaxel. [file MOL2-9999-0-s006.tif]

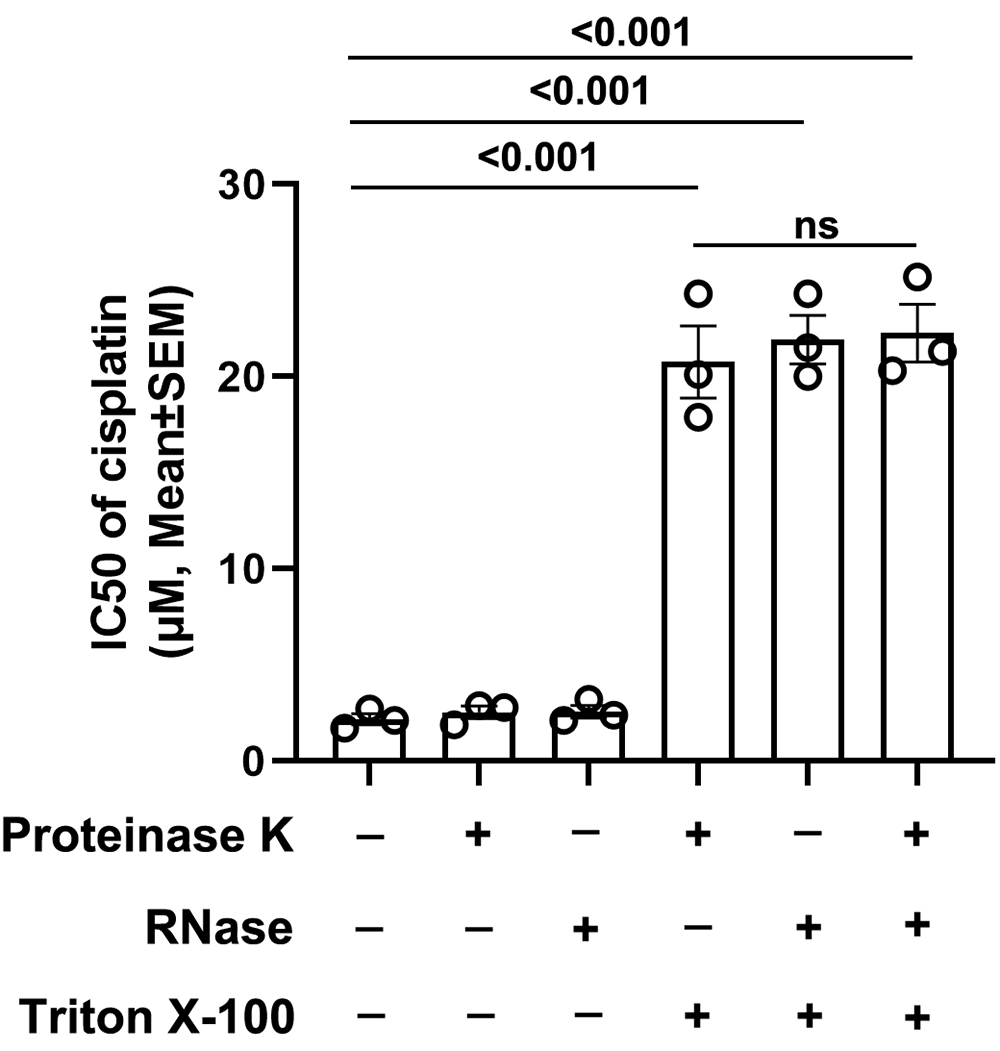

Supplement: Supplementary file 6 — Fig. S6. Vesicle integrity is required for m214‐sEV–mediated sensitization to cisplatin. [file MOL2-9999-0-s001.tif]

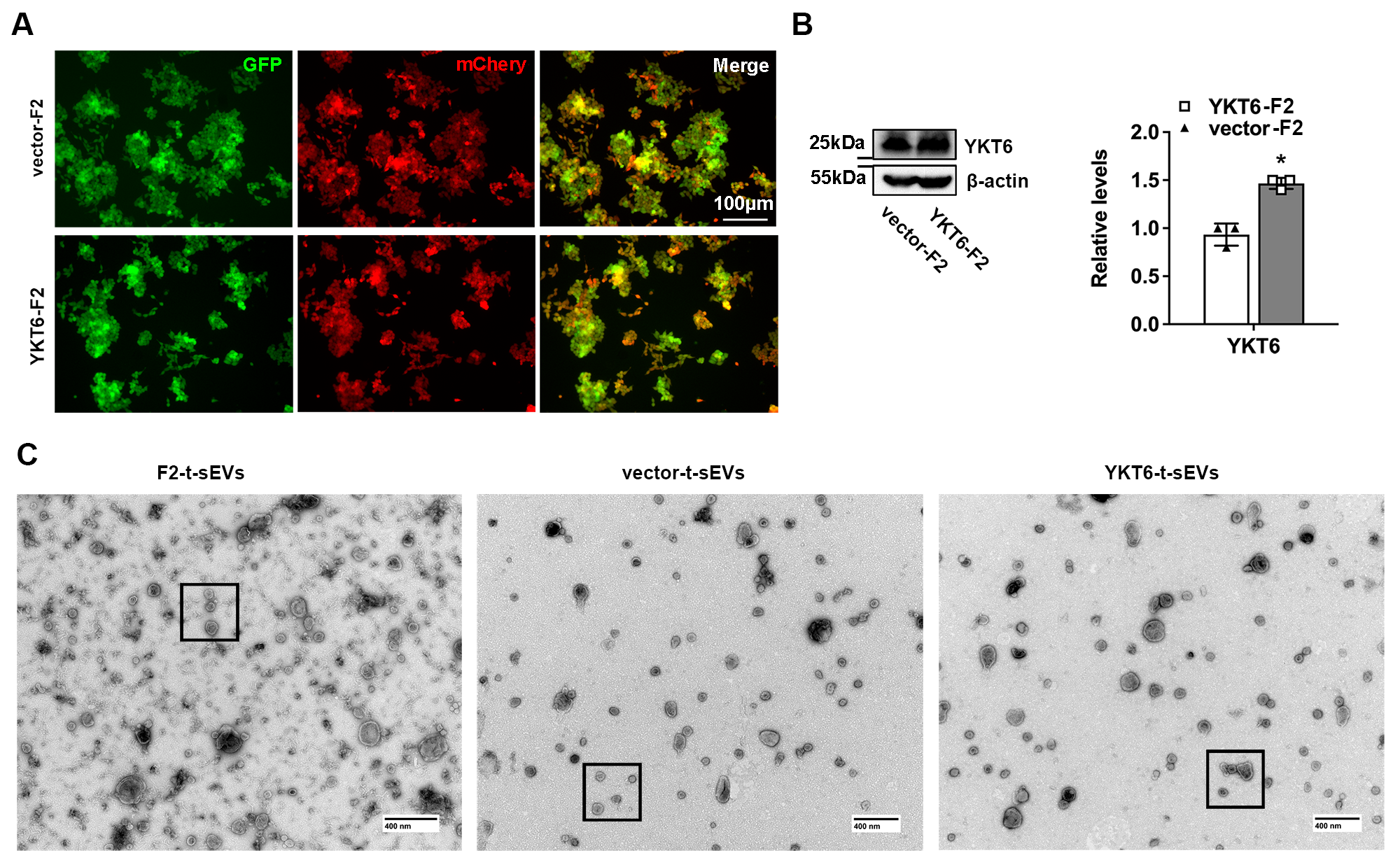

Supplement: Supplementary file 7 — Fig. S7. YKT6 overexpression in OCSC1‐F2 cells and TEM overview of derived t‐sEVs. [file MOL2-9999-0-s009.tif]
