# Supplementary material for: Engineered extracellular vesicles enriched with the miR‐214/199a cluster enhance the efficacy of chemotherapy in ovarian cancer
Source: Mol Oncol. 2026 Feb 13:10.1002/1878-0261.70224. Online ahead of print. doi: 10.1002/1878-0261.70224 (PMC13398902; doi:10.1002/1878-0261.70224)
Supplement: Supplementary file 8 — Table S1. Patient demographics of the study population stratified by OC grade. [file MOL2-9999-0-s010.docx]

**Supplementary figure legends**

**Supplementary Figure 1. In situ detection of miR-214-3p and miR-199a-5p reveals stromal enrichment in recurrent HGSOC.**

(**A**) The H&E-stained section of a representative HGSOC sample of recurrent disease (ID# SKI22475) illustrates demarcated tumor and stromal regions. (**B**) Bright-field images of the selected area from (**A**) following miRNAscope in situ hybridization (ISH) for miR-214-3p and miR-199a-5p, with U6 as a positive control. (**C, D**) Higher-magnification views of the boxed regions in (**B**) showed low expression of miR-214-3p and miR-199a-5p in tumor cells, with stronger signals observed in adjacent stromal compartments. Scale bars in A, 200 µm, B, 100 µm, C, 20 µm.

**Supplementary Figure 2. Immunohistochemical characterization of primary and recurrent ovarian cancer tissues.**

(**A**) Representative H&E staining and IHC images of primary (W4) and recurrent (RW4) ovarian cancer tumor tissues stained for the ovarian cancer stem cell marker CD44, the EMT marker vimentin, and the activated fibroblast marker α-SMA. Low-magnification images illustrate overall tumor architecture, with boxed regions shown at higher magnification to highlight marker expression patterns. Scale bars, 100 µm.

(B) Semiquantitative scoring of CD44, vimentin, and α-SMA immunoreactivity in primary and recurrent OC tissues (n = 5 tumors per group). Recurrent tumors exhibit significantly increased CD44 and vimentin expression compared with primary tumors, whereas α-SMA expression remains unchanged. Data are presented as mean ± SEM. ** P < 0.01, **** P < 0.0001; ns, not significant, assessed by two-way ANOVA.

**Supplementary Figure 3. Single EV profiling using the ExoView system**.

Representative fluorescence images acquired using the ExoView R100 platform showed specific capture and labeling of EV particles on chips coated with antibodies against the tetraspanin markers CD63, CD81, and CD9. Strong signal intensities were observed for CEC-sEVs, scra-sEVs, and m214-sEVs on the capture spots. In contrast, minimal fluorescence was detected on control spots coated with mouse IgG (mIgG), confirming the specificity of EV capture and marker detection. Scale bars, 10 µm.

**Supplementary Figure 4. Top 20 enriched pathways associated with proteins in naïve CEC-sEVs and m214-sEVs.**

Pathway enrichment analysis was performed using the Gene Ontology (GO) Molecular Function 2025 database to identify functional categories associated with proteins detected in CEC-sEVs and m214-sEVs. The top 20 pathways were ranked by statistical significance, expressed as −log₁₀ of the adjusted P-values. The raw data and results are listed in Supplementary tables 2 and 5**.**

**Supplementary Figure 5. m214-sEVs sensitize chemoresistant OC cells to cisplatin and paclitaxel.** (**A**) IC₅₀ values of cisplatin in A2780cis cells treated with increasing concentrations of sEVs (3×10⁷, 3×10⁸, or 3×10⁹ particles/mL) in combination with cisplatin (n=3/group). Statistical analysis was assessed by one-way ANOVA with Tukey’s post hoc test. (**B**) Quantitative analysis of cytotoxicity in chemoresistant OCSC lines R182 and R2615, treated with paclitaxel (Pac; 20 µM) in combination with sEVs (3×10⁸ particles/mL) over 72 hours. Cell death was assessed using real-time CellTox Green assays. Data are presented as mean ± SEM from n = 3 wells per group. *p < 0.05, **p < 0.01 by one-way ANOVA with Tukey’s post hoc test. (**C**) Representative fluorescence images of OCSC-R182 and OCSC-R2615 cultures captured 72 hours post-treatment. Dead cells were indicated by green fluorescence. Treatment groups included paclitaxel alone, paclitaxel combined with scra-sEVs, and paclitaxel combined with m214-sEVs. Scale bars = 500 µm. (**D, E**) Western blot (**D**) and quantitative analysis (**E**) of TLR4 and β-catenin expression in OCSC-R182 cells following indicated treatments. (**F**) Caspase-3/7 activity assay in OCSC1-F2 cells showed increased apoptosis following combined treatment with m214-sEVs and paclitaxel (Pac) (n=3/group). *p < 0.05 vs. Pac or other treatment groups, assessed by one-way ANOVA. Data shown in (D-F) are representative of three independent experiments.

**Supplementary Figure 6. Vesicle integrity is required for m214-sEV–mediated sensitization to cisplatin.**

The role of vesicle integrity in m214-sEV–mediated cisplatin sensitization was evaluated in A2780cis cells. m214-sEVs were pretreated with Proteinase K or RNase, with or without the membrane-disrupting detergent Triton X-100, prior to application on A2780cis cells. Subsequent measurement of cisplatin IC₅₀ values revealed that intact m214-sEVs significantly reduced cisplatin resistance.  This sensitizing effect was abolished when vesicle membranes were disrupted by Triton X-100, indicating that structural integrity is critical for functional delivery. Data represent mean ± SEM (n = 3). P values (<0.001); ns, not significant, were assessed by one-way ANOVA.

**Supplementary Figure 7. YKT6 overexpression in OCSC1-F2 cells and TEM overview of derived t-sEVs.**

(**A**) Representative fluorescence microscopy images showed co-expression of GFP (vector marker) and mCherry (cell label) in both vector-F2 and YKT6-F2 cells, confirming successful transduction. (**B**) Western blot analysis and corresponding quantification demonstrated significantly elevated YKT6 protein levels in YKT6-F2 cells compared to vector-F2 controls. *p < 0.05 by unpaired t-test (n = 3). (**C**) Low-magnification TEM images showed the morphology of tumor-derived sEVs (t-sEVs) isolated from parental OCSC1-F2 cells, vector-F2 cells, and YKT6-F2 cells. Boxed areas corresponded to regions magnified in **Fig. 6H.** Data are representative of three independent experiments. Error bars in B indicate SEM. Scale bars in A, 100 µm; C, 400 nm.

**Supplemental Table 1** Patient demographics of the study population stratified by OC grade.

| **Variables** |  | **G1+G2**  **N = 62(%)** | **G3+G4**  **N = 425 (%)** |
| --- | --- | --- | --- |
| **Age at Diagnosis** | <55 | 29 (46.8) | 144 (33.6) |
|  | >=55 | 33 (53.2) | 281 (65.6) |
| **Tumor Descriptor *** | primary | 62 (100.0) | 425 (100.0) |
|  | recurrence | 7 (11.3) | 4 (0.9) |
| **Treatment** | chemotherapy | 56 (90.3) | 395 (92.1) |
|  | hormone therapy | 4 (6.5) | 34 (7.9) |
|  | immunotherapy | 5 (8.2) | 11 (2.6) |
|  | targeted molecular therapy | 12 (19.4) | 28 (6.5) |
|  | radiation therapy, NOS | 56 (90.3) | 407 (94.9) |
| **Vital Status **** | alive | 23 (37.1) | 166 (38.7) |
|  | dead | 39 (62.9) | 263 (61.3) |

* Describes the kind of disease present in the tumor specimen as related to a specific timepoint.

**The survival state of the person registered on the protocol.

NOS = Not Otherwise Specified.

**Supplemental Table 4** Cox proportional hazards model for survival analysis.

| **mir-214-3p** | | |
| --- | --- | --- |
| HR | 95% CI | P-value |
| 0.192 | 0.059 - 0.628 | 0.0144 |
|  |  |  |
| **mir-199a-5p** | | |
| HR | 95% CI | P-value |
| 0.32 | 0.093 - 1.097 | 0.0489 |
